# Supplementary material for: Disrupted hippocampal theta-gamma coupling and spike-field coherence following experimental traumatic brain injury
Source: eLife. 2026 Jun 12;13:RP100642. doi: 10.7554/eLife.100642 (PMC13263039; doi:10.7554/eLife.100642)
Supplement: Peer review history

# Editor report: eLife Assessment

**DOI:** 10.7554/eLife.100642.3.sa0

**Authors:** Lisa M Giocomo

**Affiliations:** Howard Hughes Medical Institute, Stanford University School of Medicine United States

**Keywords:** Convincing, Important

This is an **important** paper that reports in vivo physiological abnormalities in the hippocampus of a rat model of traumatic brain injury (TBI). In this study, authors focused on changes in theta-gamma phase coupling and action potential entrainment to theta, phenomena hypothesized to be critical for cognition. The authors provide **convincing** evidence of deficits in both features post-TBI and contributes new understanding to how disruptions in oscillatory coordination and spike timing may relate to cognitive impairment.

# Referee report: Reviewer #1 (Public review)

**DOI:** 10.7554/eLife.100642.3.sa1

Summary:

This study examines how traumatic brain injury (TBI) alters hippocampal network dynamics and single-unit activity in awake, behaving rats. Using laminar recordings, the authors report reductions in theta power, theta-gamma phase-amplitude coupling, and spike-field entrainment, alongside impairments in spatial memory performance.

Strengths of the study include the use of high-density laminar electrodes to localize activity across hippocampal layers and the integration of electrophysiological and behavioral measures. Analyses that consider behavioral state and account for broadband power changes improve confidence in the interpretation of oscillatory effects. Additional controls suggest that the observed differences are unlikely to be explained by gross motor or motivational deficits. The reported relationships between theta amplitude, phase-amplitude coupling, and spike entrainment provide useful insight into how network coordination is disrupted following injury.

There are a few minor weaknesses. The analyses of single-unit activity across environments are relatively limited, and more comprehensive approaches to characterizing spatial coding would strengthen conclusions about how TBI impacts hippocampal representations. The behavioral assessment relies primarily on a single task, which constrains the interpretation of the cognitive deficits. In addition, the relatively small number of animals is a limitation, although this is partially mitigated by the number of recorded units and the consistency of effects across measures.

Overall, this work provides a careful characterization of hippocampal circuit dysfunction following TBI and contributes to understanding how disruptions in oscillatory coordination and spike timing may relate to cognitive impairment.

Comments on revisions:

The authors have adequately addressed all of my concerns.

# Referee report: Reviewer #3 (Public review)

**DOI:** 10.7554/eLife.100642.3.sa2

Summary:

In this study, authors studied the effects of traumatic brain injury created by LFPI procedure on the CA1 at network level. The major findings in this study seem to be that the TBI reduces theta and gamma powers in CA1, reduces phase-amplitude coupling in between theta and gamma bands as well as disrupts the gamma entrainment of interneurons. I think the authors have made some important discoveries that could help advance the understanding of TBI effects at physiological level, however, more investigations into deciphering the relationship of the behavioral and brain states to the observed effects would help clarify the interpretations for the readers.

Strengths:

The authors in this study were able to combine behavioral verification of the TBI model with the laminar electrophysiological recordings of CA1 region to bring forward network level anomalies such as the temporal coordination of network level oscillations as well as in the firing of the interneurons. Indeed, it seems that the findings may serve future studies to functionally better understand and/or refine the therapies for the TBI.

Weaknesses:

Discoveries made in the paper and their broad interpretations can be helped with further characterization and comparison among the brain and behavioral states both during immobility and movement. The impact of brain injury in several parts of the brain can alter brain wide LFP and/or behavior. The altered behavior and/or LFP patterns might then lead to reduced spiking and unreliable LFP oscillations in the hippocampus. Hence, claims made in abstract such as "These results reveal deficits in information encoding and retrieval schemes essential to cognition that likely underlie TBI-associated learning and memory impairments, and elucidate potential targets for future neuromodulation therapies" does not have enough evidence in testing whether the disruptions were information encoding and retrieval related or due to sensory-motor and/or behavioral deficits that could also occur during TBI.

Movement velocity is already known to be correlated to the entrainment of spikes with the theta rhythm and also in some cases with the gamma oscillations. So, it is of importance to disentangle the differences in behavioral variables and the observed effects. As an example, the author's claims of disrupted temporal coding (as shown in the graphical abstract) might have suffered from these confounds. The observed results of reduced entrainment might on one hand be due to the decreased LFP power (induced by injury in different brain areas) resulting in altered behavior and/or the unreliable oscillations of the LFP bands such as theta and gamma, rather than memory encoding and retrieval related disruption of spikes synchrony to the rhythms, while on the other hand they may simply be due to reduced excitability in the neurons particularly in the behavioral and brain state in which the effects were observed, rather than disrupted temporal code. Hence, further investigations into dissociating these factors could help readers mechanistically understand the interesting results observed by the authors.

Comments on revisions:

The authors have substantially improved the manuscript in response to the previous reviews. In particular, the revisions addressing the issue of behavioral deficits that could be caused due to the TBI, which were surprisingly not present (if anything minimal) in the injured rats, have strengthened the study and improved the support for the main conclusions. Overall, the manuscript is now clearer and more rigorous. Authors have also addressed all the minor points raised in the study. As a result, the study is now solid, with the major findings broadly supported by the data.

# Author comment: Author response

**DOI:** 10.7554/eLife.100642.3.sa3

**Authors:** Christopher D Adam, Ehsan Mirzakhalili, Kimberly G Gagnon, Carlo Cottone, John D Arena, Alexandra Ulyanova, Victoria E Johnson, John A Wolf

**Affiliations:** Center for Brain Injury and Repair, Department of Neurosurgery, University of Pennsylvania Philadelphia United States; University of Pennsylvania Philadelphia United States; Center for Brain Injury and Repair, Department of Neurosurgery, University of Pennsylvania Philadelphia United States; Center for Brain Injury and Repair, Department of Neurosurgery, University of Pennsylvania Philadelphia United States; Center for Brain Injury and Repair, Department of Neurosurgery, University of Pennsylvania Philadelphia United States; University of Pennsylvania Philadelphia United States; Center for Brain Injury and Repair, Department of Neurosurgery, University of Pennsylvania Philadelphia United States; Center for Brain Injury and Repair, Department of Neurosurgery, University of Pennsylvania Philadelphia United States

The following is the authors’ response to the original reviews.

**eLife Assessment**

This is an important paper that reports in vivo physiological abnormalities in the hippocampus of a rat model of traumatic brain injury (TBI). In this study, authors focused on changes in theta-gamma phase coupling and action potential entrainment to theta, phenomena hypothesized to be critical for cognition. While the authors provide solid evidence of deficits in both features post-TBI, the study would have been stronger with a more hypothesis-driven approach and consideration of alterations of the animal's behavioral state or sensorimotor deficits beyond memory processes.

We would like to thank the reviewers for their comments on our manuscript. By incorporating their feedback, we were able to make our hypotheses more clear, expand our analyses to compare physiological processes across similar behavioral states, and address extra hippocampal input and potential sensorimotor confounds in our data.

Specifically, we have added new data in Figure 5 showing how theta amplitude correlates with theta-gamma PAC and entrainment strength. We have also added supplementary Figure 1 demonstrating that there are no differences in exploration or movement velocity in injured animals compared to shams. Supplementary Figures 2, 3, and 4 were added to compare oscillatory power while animals were still, moving at a higher velocity, and following a broadband power shift correction respectively. We also added Supplementary Figure 7 demonstrating that there were no differences in firing rates between sham and injured animals while they were still or moving and Supplementary Figure 8 showing no changes in pyramidal cell bursting. Finally, we added Supplementary Figure 10 showing that there was no difference in velocity or distance traveled during testing in the MWM between sham and injured animals and that learning curves were similar across groups before sham/injury surgery. We believe that the addition of this data significantly improves our manuscript by more strongly controlling for the animal’s behavioral state in our analyses and provides strong evidence that significant sensory/motor deficits were not present in injured animals at this injury level and time point post injury. Below we address specific points raised by the reviewers.

**Reviewer #1 (Public review):**

Summary:

This study investigated how traumatic brain injury affects oscillatory and single-unit hippocampal activity in awake-behaving rats.

Strengths:

The use of high-density laminar electrodes enabled precise localization of recording sites. To ensure an unbiased, rigorous approach, single-unit analysis was performed by a reviewer who was blind to experimental conditions. A proof of concept study was undertaken to characterize the pathology that resulted from the specific TBI model used in the main study. There was an effort to link abnormalities in hippocampal activity to memory disruption by running a cohort of rats on the Morris Water Maze task.

Weaknesses:

The paper is written as if the experiment was exploratory and not hypothesis-driven despite the fact that there is a wealth of experimental evidence about this TBI model that could have informed very specific predictions to test a hypothesis that is only hinted at in the discussion. The number of rats used for the spatial working memory experiment is not reported. Some of the statistics are not completely reported. It is also unclear what the rationale was for recording single units in a novel and familiar environment. Furthermore, this analysis comparing single-unit activity between familiar and novel environments is quite rudimentary. There are much more rigorous analyses to answer the question of how hippocampal single-unit firing patterns differ across changes in environments. There are details lacking about the number of units recorded per session and per rat, all of which are usually reported in studies that record single units. Spatial working memory assessment is delegated to a single panel of a supplementary figure. More importantly, there is no effort to dissociate between spatial working memory deficits and other motor, motivational, or sensory deficits that could have been driving the lower "memory score" in the experimental group.

In order to address these important concerns, we have made the following changes:

(1) We have updated the results section to include more rationale for the recordings and analyses used to clarify our hypotheses. In addition, we hope that our extensive characterization will lay the groundwork to inform future studies investigating circuit-specific disruptions following TBI and neuromodulatory therapies.

(2) The number of rats used for the spatial working memory experiment is reported in the text and figure legend.

(3) We have added supplemental Table 2 to include the requested statistical information (t-statistic, degrees of freedom, and 1 vs 2-tailed analyses).

(4) Unfortunately, we did not have adequate occupancy to robustly extract and compare place cell properties across groups and environments which obscured the rationale of our study design and limited us to more rudimentary analyses. While animals did actively explore the two environments, the relatively short recording time limited the spatial sampling of the two-dimensional environment. We were able to extract putative place cells and found some evidence that place cells in TBI rats had lower spatial information content than in shams (as has previously been described). However, we did not feel that place cell analyses were rigorous enough to include in this manuscript due to the limited spatial sampling. Future studies in the lab will assess how TBI affects place cell information content, stability, and phase precession with better occupancy.

(5) We have added Supplemental Table 1 that includes the total number of units recorded for each animal.

(6) The spatial working memory deficit we report in the MWM is not a novel finding in this model of TBI. However, we wanted to ensure that _L_FPI in our hands at this injury level reproduced this known deficit. Importantly, the swim speed and distance traveled during testing did not differ between groups, suggesting that differences were not due to motor deficits. Additionally, the learning curves before sham/_L_FPI surgery were the same across groups. This data has been added to the manuscript in Supplementary Figure 10. While we did not test animals in a version of the task where the platform was visibly marked, previous studies have demonstrated that sham and injured rats perform comparably in a version of the MWM where the platform is visible or when a constant start location is used. These citations have been added to the manuscript.

**Reviewer #1 (Recommendations for the authors):**

For a more rigorous way of analyzing changes in hippocampal firing patterns across environments, see Wills et al 2005 for example.

Addressed in point 4 above

Spatial working memory tasks should always be compared with a control task to rule out confounding performance variables. Examples would be to use a variant of the MWM task that does not require the hippocampus such as using a visible escape platform.

Addressed in point 6 above

Statistics are typically reported including a t-statistic and degrees of freedom, not just the p-value. In addition, the authors should indicate whether the t-test is one or two-tailed.

Addressed in point 3 above

**Reviewer #2 (Public review):**

Summary:

The authors investigate changes in theta-gamma phase-amplitude coupling, and action potential entrainment to theta following traumatic brain injury (TBI). Both phenomena are widely hypothesized to be important for cognition, and the authors report deficits in both after TBI. The manuscript is well-written, the figures are well-constructed, and the author's use of high-level analysis methods for TBI EEG data collected from awake, behaving animals is welcome.

Major Comments:

The animal n's are small (4 sham and 5 injured). In Figure 3, for instance, one wonders if panels D and E might have shown significant differences if more animals had been recorded.

There are conflicting reports regarding the effect of _L_FPI on single cell firing rates. This is likely due to differential task demands and variations in _L_FPI severity across studies. We agree that the firing rates do appear to be trending; however, overall firing rate changes can be difficult to interpret. Because firing rates are influenced by behavior and brain state, we further separated firing rates into epochs when animals were moving or still and found similar trends that did not reach significance (data added in Supplementary Figure 7). We also assessed bursting in pyramidal cells to investigate whether potential changes in bursting influenced overall firing rates, and we found no differences between sham and injured animals across conditions (data added in Supplementary Figure 8). While the n’s are small when considered by animal, the number of units is actually fairly large, so if there were robust effects (as there were for the entrainment analyses), we would expect to see significant differences.

The text focuses on deficits in the theta and gamma bands, but the reduction in power appears to be broadband (see Figure 1F, especially Pyramidal cell layer panel). Therefore, the overall decrease in broadband (in the injured population) must be normalized between sham and injured animals before a selective comparison between sham and injured animals can be conducted. That is the only way that selective narrow bands i.e., theta and low gamma can be compared between the two cohorts. A brief discussion of the significance of a broadband decrease would be appreciated.

This is an excellent point that has now been addressed with the addition of Supplementary Figure 4. We used a well-established method (Donoghue et al 2020) to flatten power spectra in order to compare specific frequency bands in the context of a broadband shift. After applying this correction, we show that theta power is still reduced in injured rats compared to shams. While there is no difference in gamma power between groups in the corrected power spectra, this result should be interpreted with caution especially since there is not a large distinct peak in the gamma frequency range in the power spectrum of either sham or injured animals. However, if this is interpreted to mean that gamma power is not different between sham and injured animals, it makes the PAC data even more compelling. While there is clearly a broadband shift, the frequency range of this shift is still limited in the frequency domain to ~4-90Hz which contains physiologically relevant frequencies associated with synaptic currents. Importantly, the power spectra of sham and injured animals converge at low (<4Hz) and high (>100Hz) frequencies. This suggests that slow oscillations which could include delta and respiration-associated oscillations are not affected by TBI (though sleep recordings would be needed to properly address this). High-frequency activity can include ripples and HFOs which need to be separately extracted when comparing between groups due to their transient nature. However, overall spiking activity including the depolarizing spike and the after hyperpolarization significantly contribute to power in the high frequency range. Because this general high-frequency power is not different between groups, it suggests that the limited range of the broadband power reduction still contains important physiological signals. This broadband shift may result from a global reduction in or desynchronization of synaptic input to CA1. The specific mechanisms behind this broadband shift and the consequences it has on coding information in the hippocampus are fascinating questions that we hope will be specifically investigated in future studies. This point is now addressed in the Discussion.

**Reviewer #2 (Recommendations for the authors):**

Minor Comments:

Please define your reference waveform for theta - is it theta recorded on the channel containing the cell? Average theta for all electrodes in SP? SP + SO? Theta for the nominal "St. pyr." channel? Please define.

For all entrainment analyses, entrainment was measured referenced to the theta oscillation recorded from *st. pyr*. on the specific shank where the unit was detected. We added clarification in the results and methods sections regarding this point.

Similarly, even though the peak of the theta wave appears from the figures to be taken as 0 degrees, please explicitly state this in the text.

This has been added to the results and methods.

Did the authors check for any difference between interneurons in SP and interneurons in SO?

This is an excellent suggestion that we had hoped to investigate as it could inform whether specific interneuron populations were affected. However, we did not record enough units in *st. ori* to make this comparison.

On page 8, Figures 3E and 3F are incorrectly labeled 4E and 4F.

This has been fixed.

Figure 1, panel C: please add a numerical scale to the colored scale bar.

This has been added

Figure 1, panel F: how was the significance between the frequency bands calculated?

Statistics were done using a t-test at each frequency point with significance set at α=0.01 for multiple comparisons. This has been clarified in the figure legend and methods.

Figure 3, panel A legend: Please add "Spike at 0 ms omitted for clarity.”

This has been added

Figure 4, panel A, right side: please provide the MVL for this cell, so that readers have a benchmark for evaluating the MVL as a parameter. A sample poorly entrained cell, with MVL, would also be informative.

We added the MVL for this cell. We were unable to add a poorly entrained cell without making the figure more confusing.

Raw data must be provided for the Morris Water Maze experiments described in Supplementary Figure 3.

We added data showing no difference in the swim velocity or distance traveled between the sham and injured groups during memory testing as well as data showing that the two groups had similar learning curves during training before sham/injury surgery. See Supplementary Figure 10.

Antibody 22C11 for APP has been shown to be non-specific when used for immunocytochemistry (it may be fine for Westerns). In addition, using a biotinylated secondary with an ABC kit for visualization risks contamination by post-injury changes in biotin. Reviewed in Xiong et al., 2023, https://www.ncbi.nlm.nih.gov/pmc/articles/PMC10580020/.

As is standard practice in neuropathology, negative controls were run for all of these experiments (identical preparations minus the primary antibody.) No non-specific staining was present that could be mis-interpreted as APP-positive axonal profiles in either sham or injured tissue. While beyond the scope of this response, there are many reasons the authors of the cited paper may have had non-specific staining, including a concentration 450X that of the one utilized here and the absence of an antigen-retrieval technique in their protocol.

Tummala et al. used in vivo calcium-imaging after TBI and also investigated single-cell activity in familiar and novel environments, and when moving or still. The authors could consider discussing their work.

We have added a citation for this paper

**Reviewer #3 (Public review):**

Summary:

In this study, the authors studied the effects of traumatic brain injury created by LFPI procedure on the CA1 at the network level. The major findings in this study seem to be that the TBI reduces theta and gamma powers in CA1, reduces phase-amplitude coupling in between theta and gamma bands as well as disrupts the gamma entrainment of interneurons. I think the authors have made some important discoveries that could help advance the understanding of TBI effects at the physiological level, however, more investigations into deciphering the relationship of the behavioral and brain states to the observed effects would help clarify the interpretations for the readers.

Strengths:

The authors in this study were able to combine behavioral verification of the TBI model with the laminar electrophysiological recordings of the CA1 region to bring forward network-level anomalies such as the temporal coordination of network-level oscillations as well as in the firing of the interneurons. Indeed, it seems that the findings may serve future studies to functionally better understand and/or refine the therapies for the TBI.

Weaknesses:

Discoveries made in the paper and their broad interpretations can be helped with further characterization and comparison among the brain and behavioral states both during immobility and movement. The impact of brain injury in several parts of the brain can alter brain-wide LFP and/or behavior. The altered behavior and/or LFP patterns might then lead to reduced spiking and unreliable LFP oscillations in the hippocampus. Hence, claims made in the abstract such as "These results reveal deficits in information encoding and retrieval schemes essential to cognition that likely underlie TBI-associated learning and memory impairments, and elucidate potential targets for future neuromodulation therapies" do not have enough evidence to test whether the disruptions were information encoding and retrieval related or due to sensorymotor and/or behavioral deficits that could also occur during TBI.

Movement velocity is already known to be correlated to the entrainment of spikes with the theta rhythm and also in some cases with the gamma oscillations. So, it is important to disentangle the differences in behavioral variables and the observed effects. As an example, the author's claims of disrupted temporal coding (as shown in the graphical abstract) might have suffered from these confounds. The observed results of reduced entrainment might, on one hand, be due to the decreased LFP power (induced by injury in different brain areas) resulting in altered behavior and/or the unreliable oscillations of the LFP bands such as theta and gamma, rather than memory encoding and retrieval related disruption of spikes synchrony to the rhythms, while on the other hand, they may simply be due to reduced excitability in the neurons particularly in the behavioral and brain state in which the effects were observed, rather than disrupted temporal code. Hence, further investigations into dissociating these factors could help readers mechanistically understand the interesting results observed by the authors.

We appreciate the Reviewer’s insights into disentangling the complex interactions between power, entrainment, and excitability, and have attempted to dissociate these further in our analyses. Regarding the broad effects of TBI, we agree that TBI affects many brain regions outside of the hippocampus as well as white matter pathways containing axons from areas where pathology is not visible, which likely results in widespread changes to LFPs across regions and altered behavior. Here we report disrupted network activity in the hippocampus which is likely a consequence of numerous pathologies across multiple brain regions. In the discussion, we speculate that disrupted power and coupling comes from desynchronization of inputs (especially those from the mEC and MS) as well as changes to local circuits within the hippocampus which combine to disrupt temporal coding. While the disrupted processes we report in the hippocampus are implicated in computational processes thought to support learning and memory, we acknowledge that results from this study do not causally reveal a specific mechanism that is directly responsible for cognitive impairments. We have changed the language of the quoted sentence from the abstract to make our claim less causal as we agree that the direct effects of these results on cognition are difficult to quantify due to the fact that animals were not performing a spatial navigation task with measurable outcomes during recordings. We have also removed the graphical abstract as we believe it is an oversimplification of the results given new analyses.

Regarding the possible contribution of sensory and motor deficits or differences in behavioral states to the observed changes, we agree that it is essential to consider potential sensorimotor deficits as well as the animal’s behavioral state when comparing oscillations and single unit activity in the hippocampus, especially since these phenomena have been extensively liked to movement velocity and exploration. To address this, we have added Supplementary Figure 1 showing that there are no differences in movement velocity or exploration time between sham and injured animals. Because animals were simply foraging during electrophysiological experiments we do not expect there to be any major additional behavioral differences that would influence oscillations or spiking once locomotion is controlled for, though differences in attention or arousal cannot be ruled out. Additionally, analyses throughout the manuscript are performed independently during periods when animals were moving or still. Data in Figures 1 and 2 also only include data from the familiar environment to rule out any effects of novelty on hippocampal oscillations. Supplementary Figures 2 and 3 were added to demonstrate that TBI-associated reductions in power were consistent when animals were still and when a higher threshold for movement (>20 cm/sec) was used. Finally, supplementary Figure 10 was added showing no differences in swim velocity or distance traveled in the MWM between sham and injured animals, further suggesting that there are no significant sensorimotor deficits at this injury level and timepoint. Additionally, previous studies have demonstrated that sham and injured rats perform comparably in a version of the MWM where the platform is visible or when a constant start location is used, which provides further support that sensorimotor deficits are not responsible for memory deficits in this task (see above).

Regarding the contribution of neuronal excitability to the reported changes, we agree that changes in the excitability of neurons could have a strong effect on entrainment. Importantly, we show that the disrupted oscillations recorded in the injured hippocampus do not coincide with significant changes in neuronal firing rates between sham and injured animals. We have added Supplementary Figure 7 demonstrating this holds true both when animals are still and when they are moving. Additionally, we have added Supplementary Figure 8 showing no differences in pyramidal cell bursting between sham and injured animals. While this suggests that there are not major changes in excitability, homeostatic plasticity mechanisms may impact firing rates and bursting, and the extent of these effects and their role on entrainment are unclear. This point was added to the Discussion.

To address the effects of LFP power on entrainment strength, Figure 5 has been updated to show theta and gamma entrainment strength as well as theta-gamma PAC as a function of theta amplitude. We found that, during periods of comparable theta power, interneurons from sham and injured animals are similarly entrained to theta, but pyramidal cells from injured animals become significantly more entrained to theta than in shams. We address the potential implications of these results in the Discussion.

**Reviewer #3 (Recommendations for the authors):**

The authors have stated on page 7 and Figure 2E, "Taken together, injured rats show a decrease in the strength of theta-gamma PAC that is specific to st. pyr, and a shift in peak gamma amplitude to a later phase of theta in both st. pyr and st. rad". Is the shift in the peak position greater than expected by chance?

We are unaware of a rigorous method that would allow us to compare this shift statistically. We have reported the observed shift and avoided calling the shift significant for that reason.

The authors state on page 9 "cells (sham familiar=1.63{plus minus}0.23 Hz, n=51, injured familiar=2.11{plus minus}0.20 Hz, n=141, p=0.446; sham novel=1.84{plus minus}0.18 Hz, n=55, injured novel=2.23{plus minus}0.21 Hz, n=134, p=0.170; mean{plus minus}SEM; ks-test; Fig 4E) between sham and injured groups, but a higher percentage of pyramidal cells were active (firing rate >0.1Hz) in both the familiar and novel environment in injured rats compared to shams (sham=74%, injured=87%, p=0.025, Fisher's exact test; Fig 4F)." Do the authors mean Figures 3E and 3F respectively in place of Figures 4E and 4F?

This has been fixed.

Regarding the finding of similar firing rates and differences in the overlap of the neurons that were active in between injured and control animals, it is imperative to study the differences in behaviors of the animals. First of all, it seems appropriate to quantify and compare the immobility and mobile periods as well as the movement velocity of the animals in both groups. Then, it would be interesting to see if any behavioral variables correlate with the firing characteristics of the cells in both the sham and the injured animals. Since hippocampal cells have been known to have different levels of recruitment and firing rates according to different behavioral states such as movement velocity, some of the similarities or differences in neural findings might as well be attributed to the differences in behaviors in between the groups. However, some differences may be observed in the injured rats despite similar behavior and the LFP powers. In other words, studying the effects of injury during similar behavioral (e.g. firing rate as a function of movement velocity) and brain states (e.g. categorical effects of awake theta state, type two theta, and ripple states on firing rates and the entrainment) might help dissociate some effects that might only be due to difference in the behavior caused by the injury throughout the brain and might as well have less to do with specific injury induced local circuits level deficits in the hippocampus. The results in Figures 4, 5, and 6 reveal such interesting differences and hence, it becomes even more important to quantify and correlate behavioral states (movement velocity and theta/ripple) to the neuronal characteristics (LFP power, PAC, firing rates, and entrainment) presented in Figure 3.

These are excellent points, and we have addressed them in the following ways:

We added Supplementary Figure 1 demonstrating that there were no differences in movement velocity between sham and injured animals during electrophysiological recordings.

Power and PAC analyses were done exclusively when the animal was moving to compare across similar behavioral states. Additionally, these analyses were constrained to recordings from the familiar environment to rule out any effects of novelty. Because animals were simply foraging during recordings we do not expect other behavioral factors besides movement velocity to play a major role in these processes. We have also added Supplementary Figures 2 and 3 which demonstrate that TBI-associated differences in oscillatory power follow similar trends when animals are still (Sup. Fig 2) or when a higher movement threshold (>20cm/sec) is used (Sup Fig 3). We also added Supplementary Figures 7 and 8 showing that there were no significant differences in firing rates or bursting while animals were still or while they were moving.

The Discussion was expanded to discuss how TBI may disrupt circuits outside the hippocampus which may contribute to our findings. Additionally, we acknowledge the limitation that these recordings were not obtained while animals were doing a quantitatively measurable spatial navigation task which limits our ability to assess whether changes are truly behaviorally relevant.

We have also updated Figure 5 to show entrainment across different levels of theta power.

Elaborating on the abovementioned point, Figures 4B and 4E depict a finding that mean entrainment is reduced in the injured during immobility. The following factors may contribute to the results:

(1) Reduction in theta power during immobility (reduced attention and/or LFP profile due to brain-wide injury), which makes theta cycles unreliable, which can contribute to the results.

(2) Changes in neural firing properties during immobility, such as reduced burst rates or firing rates during immobility.

(3) As the authors claimed in the graphical abstract, there might be an actual disruption of temporal code associated with the memory encoding. It would be awesome if the temporal disruption could be investigated during the comparable theta power and behavioral states. This analysis would test whether there is an unconfounded disruption in the temporal code in the hippocampus due to the injury. In any case, it would be ideal to isolate the epochs during sleep in which animals were in theta state and exclude ripple states to make a definitive assessment of the aforementioned factors. These further investigations would also help the interpretations made by authors in the discussion section such as "This can disrupt type II theta which occurs when animals are not actively moving and exploring the environment. We found that single unit entrainment to theta was substantially decreased in injured rats when they were not moving, a phenomenon not seen in shams, which suggests a disruption in type II theta. This provides further evidence that cholinergic signaling may be dysfunctional following TBI."

(1) While theta power is reduced in injured animals, it can still be reliably detected even at rest. We added Supplementary Figure 2 showing power spectra while animals were not moving, and a distinct peak can be seen in the theta frequency range. Additionally, clear peaks in entrainment can be seen in the theta frequency band in Fig 4B while animals were still. This suggests that theta can still be reliably detected in injured animals even when they are not moving. However, we agree that reduced attention or arousal could contribute to these changes, and this point has been added to the Discussion.

(2) We added Supplementary Figures 7 and 8 showing no differences in firing rates or bursting parameters between groups during periods of immobility.

(3) We updated Figure 5 which now shows entrainment strength as a function of theta amplitude. We found that the theta entrainment strength of both pyramidal cells and interneurons increased with increasing theta amplitudes. We address potential implications of these changes in the Discussion.

On page 10 the authors state, "theta entrainment strength drastically increased when rats began moving in injured but not sham animals." It is unclear if the effect was confined to the periods when rats started movement. Also, it would be of interest to investigate whether movement epochs and velocity were affected in the periods when the effects were observed.

This was not confined to the exact points when the rats started moving. We removed the word “began” for clarity. See point regarding velocity above.

On page 12 the authors state, "On test day, injured rats had a lower memory score than shams (sham=114.8 {plus minus} 21.8, n=9; injured=51.5{plus minus}6.8, n=14; p=0.020; mean {plus minus} SEM; Welch's t-test) indicating poor spatial memory (Sup Fig 3A)." The result is the validation of the TBI injury on a hippocampal-dependent Morris water maze task. However, it would be nice to see the quantification of the movement velocity in the water maze and the trajectory length in each group to further dissect whether animals were constrained in the movement and hence, they could not get to the platform or they forgot where it was located. Also, it would help to compare the rats' performance after sham or TBI surgeries to their performance during the training before the surgeries (assuming the data during the training periods were recorded as well).

We have added Supplemental Figure 10 to include all of this information. Importantly, movement velocity and distance traveled were not different between groups on testing day, and the learning curves of both groups were the same before sham/injury surgery.
